# Follicular dendritic cell sarcoma in the right chest wall: A case report

**DOI:** 10.1097/MD.0000000000021935

**Published:** 2020-08-28

**Authors:** Hongli Xu, Bin Chen, Chengwei Jiang, Zhaoying Yang, Keren Wang

**Affiliations:** a Department of Breast Surgery; b Department of Nuclear Medicine; c Department of Pathology, China-Japan Union Hospital of Jilin University, Changchun, Jilin, P.R. China.

**Keywords:** chest wall, ^18^F-fluorodeoxyglucose positron emission tomography/computed tomography, follicular dendritic cell sarcoma, immunohistochemistry, local tumor resection

## Abstract

**Rationale:**

Follicular dendritic cell sarcoma (FDCS) is a rare malignant tumor that originates from germinal center follicular dendritic cells, and can occur at both nodal and extranodal sites. There are very few described cases of FDCS arising in the chest wall.

**Patient concerns:**

A 44-year-old male patient presented with a history of right chest wall pain for 5 months.

**Diagnoses:**

Positron emission tomography/computed tomography showed a significant increase in ^18^F-fluorodeoxyglucose uptake and multiple small axillary lymph nodes without hypermetabolic lesions. Immunohistochemistry results of a core-needle biopsy indicated FDCS, which was consistent with the postoperative pathological examination.

**Interventions:**

The patient underwent tumor resection with lymphadenectomy of level I axillary nodes. No metastasis in the lymph nodes was observed in the postoperative pathological examination. The patient did not accept chemotherapy or radiotherapy.

**Outcomes:**

After 18 months, the patient remains in good condition with no evidence of disease recurrence.

**Lessons:**

This report highlights a rare case of a FDCS arising in the chest wall. Accurate clinical diagnosis and staging of this rare malignant sarcoma is essential for the developmnt of effective treatment strategies. Preoperative ^18^F-fluorodeoxyglucose positron emission tomography/computed tomography scanning combined with core-needle biopsy could provide differentiation between benign and malignant tumors, as well as lymph node involvement and metastatic status.

## 1 Introduction

Follicular dendritic cell sarcoma (FDCS), also known as dendritic cell sarcoma with unclear pathogenesis, is a relatively rare malignant tumor originating from follicular dendritic cells (FDC) at both nodal and extra-nodal sites FDC.^[1]^ FDC are located in the germinal centers of primary and secondary lymphoid follicles and play an important role in antigen presentation to B cells to promote their maturation.^[2]^ Seventy percent of FDCS tumors occur in mediastinal or axillary lymph nodes.^[3]^ The remaining 30% develop in other areas, such as the thyroid, breast, retroperitoneum, oral cavity, nasopharynx, parotid gland, lung, liver, spleen, pancreas, gastrointestinal tract, and intracranial areas.^[1]^ FDCS arising from the chest wall is extremely uncommon and only 2 cases have been reported to date.^[4,5]^

Diagnosis of FDCS is challenging and it is often misdiagnosed, even by oncology experts. There is not a well-designed treatment for this rare tumor and the role of adjuvant therapy remains unclear. Histopathology and diagnostic imaging play an important role in the evaluation of patients with FDCS. FDCS is of mesenchymal origin and expresses several positive markers, including CD21, CD23 and CD35, but shows negligible staining with S-100. In previous studies, ^18^F-fluorodeoxyglucose position emission tomography/computed tomography (^18^F-FDG PET/CT) has been used for biopsy guidance, treatment response evaluation, follow up, and prognostication of sarcomas. Moreover, the standard uptake value max (SUVmax) value of PET/CT (positron emission tomography/computed tomography) correlates with tumor grade.^[6,7]^ The effectiveness of ^18^F-FDG PET/CT and PET/CT in the diagnosis or development of treatment strategies for FDCS has not been well studied, due to the low number of cases.

We report here a rare case of FDCS originating in the superficial right chest wall.^18^F-FDG PET/CT and analysis of immunohistochemical (IHC) features of a core-needle biopsy were beneficial for the diagnosis, establishment of clinical staging, and development of an effective treatment strategy.

## 2 Case presentation

A 44-year-old male patient presented complaining of persistent dull pain over the right chest wall area that had been ongoing for 5 months and worsening for 1 month without other associated symptoms. A general systemic examination was normal except for a large mass on the right lateral chest wall near the axillary area. The patient had no history of hypertension, diabetes, tuberculosis, hepatitis, or other infectious diseases. He reported no history of surgery, trauma, blood transfusions, and no food or drug allergies. All routine blood tests, including a complete hemogram, renal function, and liver function were normal, and the patient tested negative for sexually transmitted diseases (hepatitis B virus, hepatitis C virus, human immunodeficiency virus, and syphilis).

**Figure 1:**
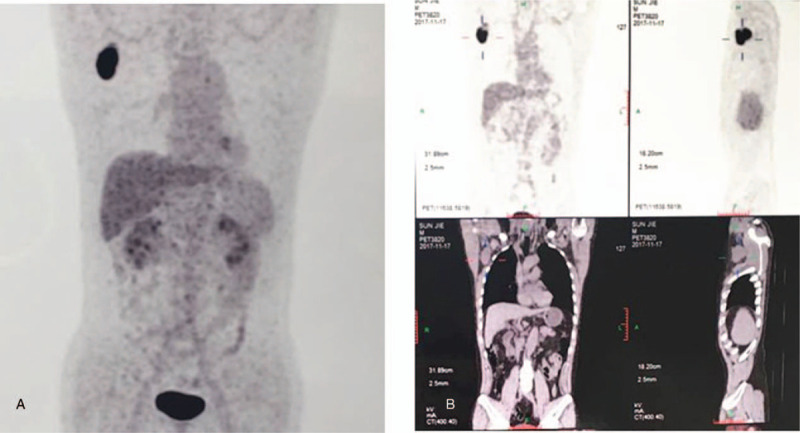
^18^F-fluorodeoxyglucose positron emission tomography/computed tomography. Increased metabolism with a standard uptake value max value of 22.82 in the right chest wall mass (A). Multiple and small lymph nodes without hypermetabolic lesions under the right axillary and right subclavian artery (B).

An ultrasound examination was performed to further evaluate the tumor in the chest wall. The tumor appeared as a bulky mass, irregular in shape, and without distinct margins. Internal blood flow signal and the arterial blood supply were measured at approximately 30.8 cm/s. Bilateral axillary lymph nodes appeared normal. ^18^F-FDG PET/CT showed a 5.2 × 4.5 × 2.8 cm^3^ soft tissue lump on the right chest wall with an increased SUVmax value of 22.82 (Fig. 1). The increased metabolism of the right chest wall mass suggested a malignant process. Multiple small lymph nodes without hypermetabolic lesions under the right axillary and subclavian artery were detected by ^18^F-FDG PET/CT. No other abnormalities or lymphadenomegaly were detected.

Results of a core-needle biopsy showed that the tumor was composed of atypical oval to spindle-shaped cells admixed with lymphoplasmacytic infiltrate. Immunohistochemistry (IHC) results revealed the following pattern: CD21 (+), CD23 (+), CD35 (+), CD163 (+), CD68 (Scat +), Vimentin (+), CK (-), TTF-1 (-), Ki-67 (30%), CD20 (B cells +), CD3 (T cells +), CD1a (-), Melan-A (-), ALK (-), and Ventana ALK (-). This constellation of findings supported a diagnosis of FDCS. The patient had no contraindications for surgery and underwent tumor resection with lymphadenectomy of level I axillary nodes.

**Figure 2:**
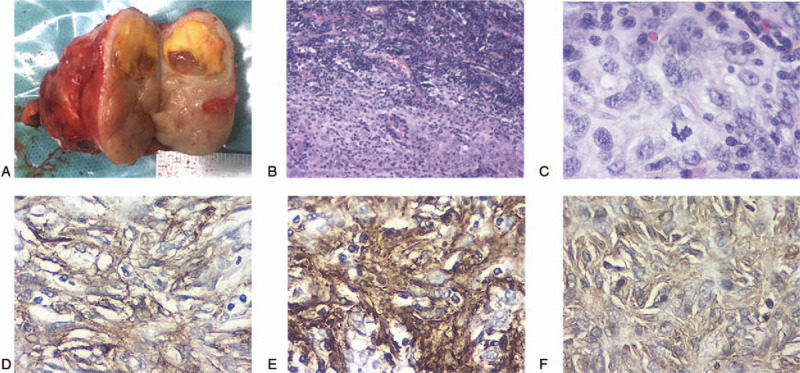
Gross and pathological images. Tumor cut-off image (A); the size of the mass was 5.5 × 4.5 × 3.0 cm^3^, and the cut surface was tan and interspersed with small and yellowish necrotic areas; the abundance of spindle cells is evident (B, 100×); spindle cell nuclear mitosis (C, 400×); immunohistochemical features (400×); positive staining for CD21 (D), CD23 (E), and Vimentin (F).

At the time of surgery, the mass had a complete capsule and could be easily separated from the surrounding tissues. The cut surface was tan to gray in color and interspersed with grossly visible hemorrhage and yellowish necrotic areas (Fig. 2A). Examination of paraffin embedded tissue samples showed destruction of the normal lymphatic structure. There was no metastasis in any of the partial axillary lymph nodes examined consistent with preoperative ^18^F-FDG PET/CT scanning. Abnormally shaped spindle cells and scattered mitotic cells were observed (Fig. 2B-C). IHC showed the following profile: CD21 (+), CD23 (+), CD68 (+), CD20 (+) Vimentin (+), Ki- 67 (20%), CD1a (-), CD3 (-), CK (-), S-100 (-), SMA (-), EMA (-) (Fig. 2D-F). This IHC profile was consistent with the patient's preoperative core-needle biopsy results, supporting a diagnosis of FDCS.

After surgery, the patient denied any self or family history of fibromatosis. The patient was treated with radical resection alone, without chemotherapy or radiotherapy. After 18 months, the patient remains in good condition with no evidence of disease recurrence.

## 3 Discussion

FDCS was first reported by Monda in 1986.^[8]^ The etiology is unclear, but some studies have suggested FDCS might be associated with Castleman disease, which is a less common non-neoplastic lymphoproliferative disease. It is reported that a few dozen of FDSC cases arising as a complication of pre-existing subtype hyaline-vascular Castleman disease.^[9]^ The most commonly reported FDCS tumor sites are in the lymph nodes or in the head and neck region, and FDCS originating from the chest wall is very rare. FDCS occurs typically in young to middle-aged patients (average age: 44 years) with no gender distinction.^[1]^ It can be easily confused with other diseases, such as inflammatory pseudotumor, angiosarcoma, hemangioendothelioma, metastatic carcinoma, and malignant fibrous histiocytoma.^[10]^ Tumor size and growth patterns are closely related to the disease site. For example, tumors located in the superficial area of the neck often show small nodular or swollen growth and a general absence of hemorrhage and necrosis.^[11]^ Tumors discovered in the abdomen tend to be deeper and larger, and are often accompanied by hemorrhage and necrosis. They may infiltrate adjacent parenchymal organs or soft tissues, and are almost always accompanied by abdominal pain.^[5,11]^ Our patient presented with an isolated mass in the right chest wall without other remarkable features clinically or radiographically.

Krober first provided PET/CT data for FDCS.^[12]^ Smith reported that glucose transporter type 1, which is associated with glucose or ^18^F-FDG uptake, was overexpressed in malignant tumors.^[13]^ Furthermore, Nakagawa reported the staining pattern for that glucose transporter type 1 and CD21 are markedly similar in lymphoid follicles, which is expressed exclusively in lymphoid follicles.^[14]^ In the current case, we found that the tumor showed extremely avid FDG uptake, with an SUVmax value of 22.82. In previous cases, the SUVmax value ranged from 4.5 to 11.4.^[15–17]^ Preoperative ^18^F-FDG PET/CT in this patient was used not only to differentiate the nature of tumor, but also to determine whether lymph nodes or organ metastasis was involved. Preoperative ^18^F-FDG PET/CT showed the mass had strong focal ^18^F-FDG uptake without hypermetabolic lesions under the right axillary and subclavian artery. Thus, the patient was subjected only to surgical resection. In this regard, it can be concluded that PET/CT examination can guide surgical management and direct other treatment measures where poor prognostic features are noted.^[6]^

Diagnosis of FDCS depends primarily on pathological examination and analysis of IHC features. The majority of FDCS are well-demarcated from the surrounding parenchyma with a solid nodular shape, yellowish necrosis, and a dust-colored surface when cut.^[18]^ The current case was relatively superficial with little bleeding, clear boundaries, and no invasion of the surrounding tissues. These findings are consistent with earlier reports of isolated FCDS cases. Upon histopathological examination, FDCS typically exhibits ovoid to spindle-shaped cells with multinucleated or mitotic cells arranged in a pattern of diffuse sheets, whorls, or fascicles, and scattered mature lymphocytes. Lymphoplasmacytic infiltration is commonly observed in tumor tissue. Saygin et al reported that lymphoplasmacytic infiltration and tumor size correlate with poor overall survival rates in FDCS patients. FDCS tumor cells show an immunophenotype similar to that of normal FDC and express 1 or more of the markers CD21, CD23, CD35, R4/23, CNA42, Ki-M4P, EMA, and CD68.^[19]^ They are typically negative for S-100, CD3, CD1a, CD30, CD34, SAM, CAM5.2, lysosomes and myeloperoxidase.^[20,21]^ Positive IHC staining for CD21, CD35, and CD23 is particularly useful for the final diagnosis of FDCS, as it can distinguish FDCS from other spindle cell neoplasms. IHC staining can also be used to differentiate FDCS from other dendritic cell tumors. For example, negative S-100 staining can differentiate FDCS from interdigitating DC sarcoma, and negative staining for CD1a can distinguish FDCS from Langerhans cell sarcoma. In this case FDCS was diagnosed by positive staining for CD21, CD23, CD68, CD20, and Vimentin, and negative staining for CD1a and S-100.

There is no standard treatment for FDCS. Many FDCS are treated as lymphomas or sarcomas based on their lymphoid or sarcomatous origin. Current treatment options include surgery, radiation therapy, and chemotherapy alone or in combination. While surgical resection is the preferred treatment plan, this is not always technically feasible in unresectable cases. Because of the lack of large clinical studies, there is no standard chemotherapy. Some investigators suggest the application of the CHOP (cyclophosphamide, doxorubicin, vincristine, and prednisolone) regimen may be effective. In recent years, the use of doxorubicin, ifosfamide, taxane, and gemcitabine for the treatment of FDCS has also gained interest. Chen et al reported a case of primary inoperable FDCS of the liver that showed no signs of progression after 8 cycles of systemic treatment with gemcitabine and docetaxel.^[22]^ Oshiro et al reported that temozolomide could inhibit the growth of doxorubicin-resistant FDCS in a patient-derived xenograft mouse model.^[23]^ Vermi et al found that activation of the epithelium growth factor receptor by cognate ligands in FDCS drove cell survival and proliferation, indicating blockage of this receptor might be clinically relevant.^[24]^ Starr et al analyzed potential DNA mutations in a thyroid FDCS and found mutations in the PTEN, RET, and TP53 genes, but not epithelium growth factor receptor.^[25]^

FDCS was once considered a low-grade sarcoma with a long course of disease. Through follow-up of treated patients in recent years, we have found that at least 40% of documented FDCS have recurred, and 25% have metastasized with a mortality rate of 16.7%.^[26,27]^ This has led to a better understanding of intermediate-grade FDCS cases. Patients with localized disease may be treated similarly to those with soft tissue sarcoma as suggested by Perkins et al.^[28]^ For patients with locally advanced or disseminated disease, it may be necessary to include postoperative chemotherapy or radiotherapy. In this case, our patient was treated with radical resection without chemotherapy or radiotherapy, and in the short-term has shown no evidence of recurrence.

## 4 Conclusions

To our knowledge, only 2 cases of FDCS in the chest wall have been reported to date. Whether ^18^F-FDG PET/CT examination before surgery can be used to generally evaluate a patient's condition and indicate appropriate treatment measures remains to be explored. Because FDCS is relatively rare and lacks special clinical and imaging features, it is typically confirmed by pathology and IHC. In the present case, the patient received a ^18^F-FDG PET/CT examination prior to surgery. This provided a reference value for differentiation between a benign and malignant tumor, evaluation of the clinical stage, and development of a treatment strategy. For FDCS, there are no definitive indicators for either surgical treatment alone or adjuvant chemotherapy and/or radiotherapy. We provide this case report, to improve preoperative examination protocols for FDCS, and to help develop appropriate treatment strategies. Because of the low incidence of this disease, it is difficult for any center to acquire adequate experience on the appropriate management of these patients.

## Author contributions

**Conceptualization:** Keren Wang, Zhaoying Yang.

**Data curation:** Hongli Xu, Bin Chen, Chengwei Jiang.

**Writing – original draft:** Hongli Xu, Bin Chen.

**Writing – review & editing:** Zhaoying Yang.
